# Strain-dependent neutralization reveals antigenic variation of human parechovirus 3

**DOI:** 10.1038/s41598-017-12458-5

**Published:** 2017-09-21

**Authors:** Eveliina Karelehto, Sabine van der Sanden, James A. Geraets, Aušra Domanska, Lonneke van der Linden, Dionne Hoogendoorn, Gerrit Koen, Hetty van Eijk, Shabih Shakeel, Tim Beaumont, Menno de Jong, Dasja Pajkrt, Sarah J. Butcher, Katja C. Wolthers

**Affiliations:** 1 Department of Medical Microbiology, Laboratory of Clinical Virology, Academic Medical Center, University of Amsterdam, Amsterdam, 1105 AZ The Netherlands; 2 Institute of Biotechnology & Department of Biosciences, University of Helsinki, Helsinki, 00014 Finland; 3 AIMM Therapeutics, Academic Medical Center, Amsterdam, 1105 AZ The Netherlands; 4 Department of Pediatric Infectious Diseases, Emma Children’s Hospital, Academic Medical Center, Amsterdam, 1105 AZ The Netherlands

## Abstract

Human parechovirus 3 (HPeV3), a member of the Picornavirus family, is frequently detected worldwide. However, the observed seropositivity rates for HPeV3 neutralizing antibodies (nAbs) vary from high in Japan to low in the Netherlands and Finland. To study if this can be explained by technical differences or antigenic diversity among HPeV3 strains included in the serological studies, we determined the neutralizing activity of Japanese and Dutch intravenous immunoglobulin batches (IVIG), a rabbit HPeV3 hyperimmune polyclonal serum, and a human HPeV3-specific monoclonal antibody (mAb) AT12-015, against the HPeV3 A308/99 prototype strain and clinical isolates from Japan, the Netherlands and Australia, collected between 1989 and 2015. The rabbit antiserum neutralized all HPeV3 isolates whereas the neutralization capacity of the IVIG batches varied, and the mAb exclusively neutralized the A308/99 strain. Mapping of the amino acid variation among a subset of the HPeV3 strains on an HPeV3 capsid structure revealed that the majority of the surface-exposed amino acid variation was located in the VP1. Furthermore, amino acid mutations in a mAb AT12-015-resistant HPeV3 A308/99 variant indicated the location for potential antigenic determinants. Virus aggregation and the observed antigenic diversity in HPeV3 can explain the varying levels of nAb seropositivity reported in previous studies.

## Introduction

Human parechoviruses (HPeVs), belonging to the *Picornaviridae* family, are an important cause of severe disease in young children. Based on nucleotide sequence diversity in the VP1 capsid protein, HPeVs are classified into 17 genotypes, of which HPeV1 and HPeV3 are the most frequently detected^1,2^. Infection with HPeVs is associated with a broad spectrum of clinical manifestations, ranging from mild gastrointestinal and respiratory symptoms to sepsis-like disease, meningitis and encephalitis in children. While most HPeVs cause mild disease in children between 1 to 5 years of age, HPeV3 infection more often leads to severe illness in infants under 3 months of age^3–5^. HPeV3 is prevalent worldwide and outbreaks have been reported in the Netherlands, Japan and Australia^1,6–11^. Despite the large clinical impact of these viruses, no vaccines or targeted antiviral therapies are available against HPeVs. Neutralizing antibodies (nAbs) have been described to be critical for protection against the closely related human enteroviruses (EVs)^12,13^. Therefore, based on the assumption that protective HPeV nAbs are present in the general adult population, intravenous immunoglobulin (IVIG) pooled from a large number of plasma donors has been used to treat severe HPeV1 infection^14^.

Details on the humoral immune response against HPeV3 and the protective role of nAbs against disease development are limited. In a seroprevalence study using *in vitro* neutralization assay against an HPeV3 strain isolated from a clinical specimen in 2006 in Finland, we previously found HPeV3 nAb seropositivity rates in Finnish and Dutch adults to be as low as 13% and 10%, respectively^15^. In line with this, a small serologic survey of adults from Wisconsin USA yielded negative results for HPeV3 neutralization^16^. In this study, an HPeV3 strain isolated in the US was used for the neutralization assays. In contrast, HPeV3 nAb seropositivity rates up to 80% were observed in the adult population in Japan^17–19^ in studies where either the prototype HPeV3 A308/99 strain or a Japanese isolate from 2008 was used in the neutralization assays. For HPeV1, neutralization rates above 90% have been reported in adults in Finland, the Netherlands and in Japan^15,18,19^. These high rates suggest that young children are likely protected against HPeV1 infection by maternal antibodies, while low prevalence of HPeV3-specific nAbs in the adult population could explain the higher rates of HPeV3-related severe illness in neonates and infants. However, the low nAb levels against HPeV3 reported in certain countries contrast with the relatively frequent detection of the virus in patients by PCR. In the Netherlands, HPeV3 infections occur biannually and, similar to HPeV1, represent approximately 3.5% of all infections reported as part of the enterovirus surveillance in those years^20^. The varying seropositivity rates of HPeV3 nAbs in different studies and the inconsistency between the nAb and the PCR detection rates may be due to the antigenic diversity among HPeV3 strains used in the serological studies. Additionally, technical aspects in serological assays may contribute to the observed differences. We have previously observed low or no neutralizing activity of homologous antiserum against the HPeV3 strain 152037, isolated from a clinical specimen in the Netherlands in 2001, in the Vero cell line, whereas efficient neutralization of the prototype HPeV3 A308/99 strain in the Vero and LLCMK2 cell lines was reported in Japan^17,21–23^. This could be due to the different cell lines and HPeV3 strains used or to virus aggregation in the cell lysates used; a phenomenon which has been shown to facilitate picornavirus escape from nAbs, and can be counteracted by chloroform treatment^24,25^.

The HPeV1 VP1 C-terminus including the receptor-binding RGD motif as well as regions of the HPeV1 VP0 and VP3 capsid proteins have been reported to be immunogenic and epitopes of two HPeV1-specific neutralizing human monoclonal antibodies (mAbs) have been characterized^26,27^. There are no neutralizing sites yet described for HPeV3. A recently resolved atomic model of HPeV3 now allows us to start mapping HPeV3 epitopes and antigenic variation to the capsid surface^28^. We studied the antigenic diversity among HPeV3 clinical isolates by characterizing the neutralizing capacity of IVIG batches from Dutch and Japanese populations, of a rabbit HPeV3 hyperimmune polyclonal serum and of an HPeV3-specific human mAb (AT12-015, AIMM Therapeutics^28^) against a panel of 25 HPeV3 isolates. To understand the genetic basis of the antigenic variation and to identify potentially immunogenic sites, we sequenced the capsid-encoding regions of 10 HPeV3 isolates, generated an AT12-015-resistant (MAR) HPeV3 variant and mapped the observed amino acid variation on the virus capsid structure.

## Results

### Chloroform treatment rendered the HPeV3 isolate 152037 susceptible for neutralization in the LLCMK2 cell line

To investigate virus aggregation, the neutralizing capacity of a rabbit HPeV3 hyperimmune polyclonal serum was determined against untreated and chloroform-treated prototype HPeV3 A308/99 and the HPeV3 152037 strain in LLCMK2, a cell line which is often used in HPeV3 studies and supports replication of both strains^18,23,29^. The untreated HPeV3 152037 strain could not be neutralized by the rabbit HPeV3 hyperimmune polyclonal serum, while a nAb titer of 11.0 log2 was observed following chloroform treatment of the virus. HPeV3 prototype A308/99 strain nAb titers were high against both untreated and chloroform treated virus, >13.0 and 16.5 log2, respectively.

### HPeV3 neutralization is strain-dependent

**Figure 1:**
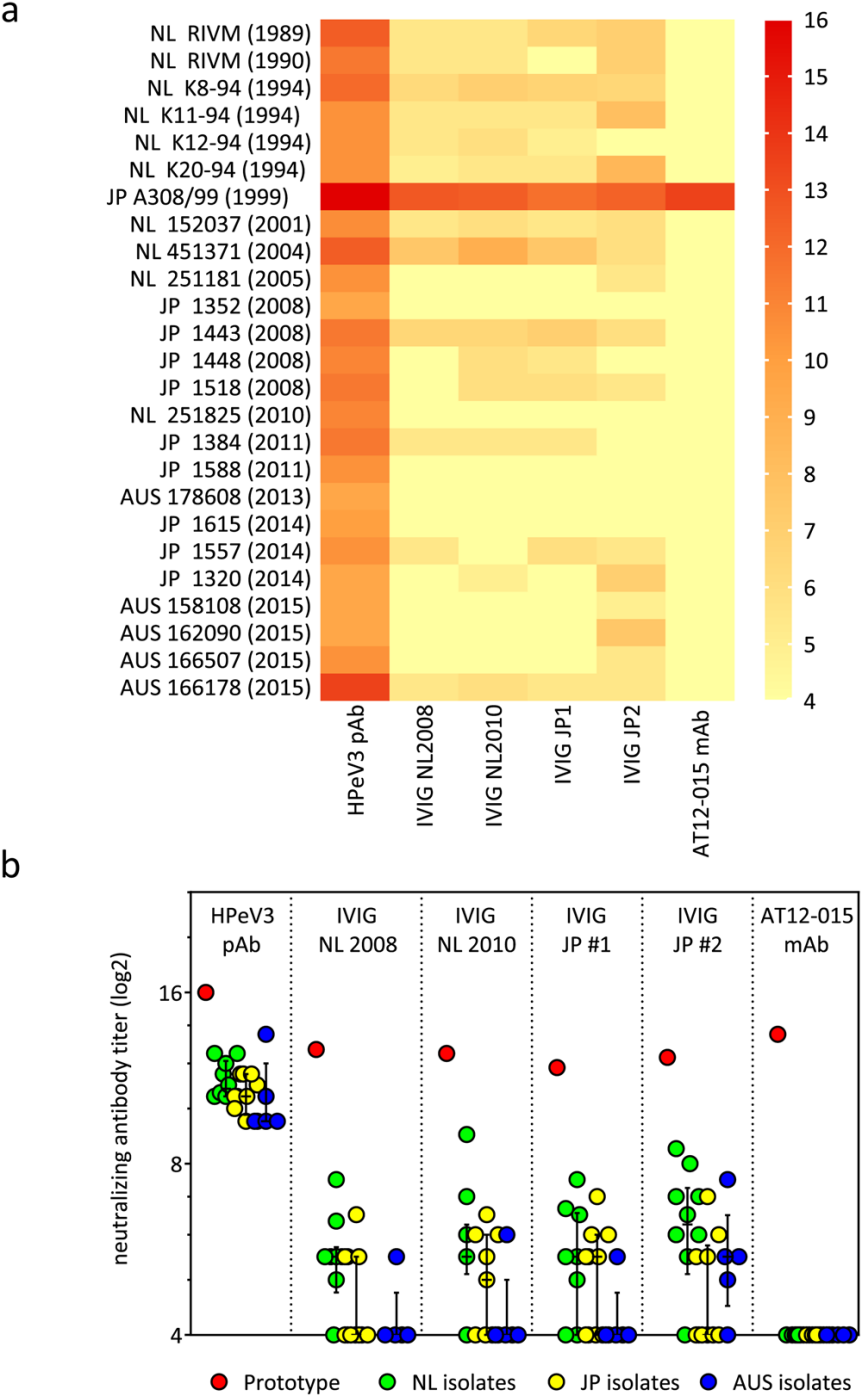
Neutralization capacity of a polyclonal rabbit HPeV3 antiserum (HPeV3 pAb), the Dutch (NL) and the Japanese (JP) IVIG batches and a human AT12-015 monoclonal antibody (HPeV3 mAb) against a panel of 25 HPeV3 isolates. (**a**) Heat map representation of log2-transformed nAb titers against individual HPeV3 isolates arranged based on year of isolation. (**b**) Comparison of prototype HPeV3 A308/99 strain nAb titers against the median nAb titers (with interquartile range) of the clinical HPeV3 isolates. Samples were grouped by the geographical location of isolation (Dutch isolates; NL, Japanese isolates; JP and Australian isolates; AUS). Positivity was defined as a titer ≥5 log2. HPeV3 isolate group median nAb titers with interquartile range were compared by Kruskal-Wallis test with Dunn’s post hoc analysis (significance level p < 0.05).

To study antigenic diversity of HPeV3, we determined the neutralizing activity of the rabbit HPeV3 hyperimmune polyclonal serum, an HPeV3-specific human mAb AT12-015, and Dutch and Japanese IVIG batches against a panel of chloroform-treated HPeV3 isolates. Figure 1a shows the nAb titers against all tested HPeV3 strains, sorted by year of isolation. The rabbit polyclonal serum neutralized all HPeV3 strains with high nAb titers ranging from 9.5 to 16.0 log2 indicating the presence of neutralizing epitopes on the capsid surface of all strains (Fig. 1a). IVIG batches from Japan and the Netherlands could neutralize the Dutch HPeV3 strains from 1989 to 2004 and the prototype strain A308/99 from Japan (1999). However, as defined by the positivity cut-off of ≥5.0 log2, low or no nAb titers were observed against the more recently isolated strains (from 2005 to 2015). Remarkably, the mAb AT12-015, isolated from a Dutch donor in 2012, exclusively neutralized the prototype A308/99.

To compare neutralization of the prototype HPeV3 A308/99 with that of the clinical isolates, nAb titers were analyzed based on the geographical location of isolation (Fig. 1b). The rabbit HPeV3 hyperimmune polyclonal serum and all IVIG batches had considerably higher nAb titers against the prototype strain than against the clinical isolates. No significant differences were found between median titers of the clinical isolate groups.

### The majority of the sequence variation is mapped to surface exposed regions of the HPeV3 capsid

**Figure 2:**
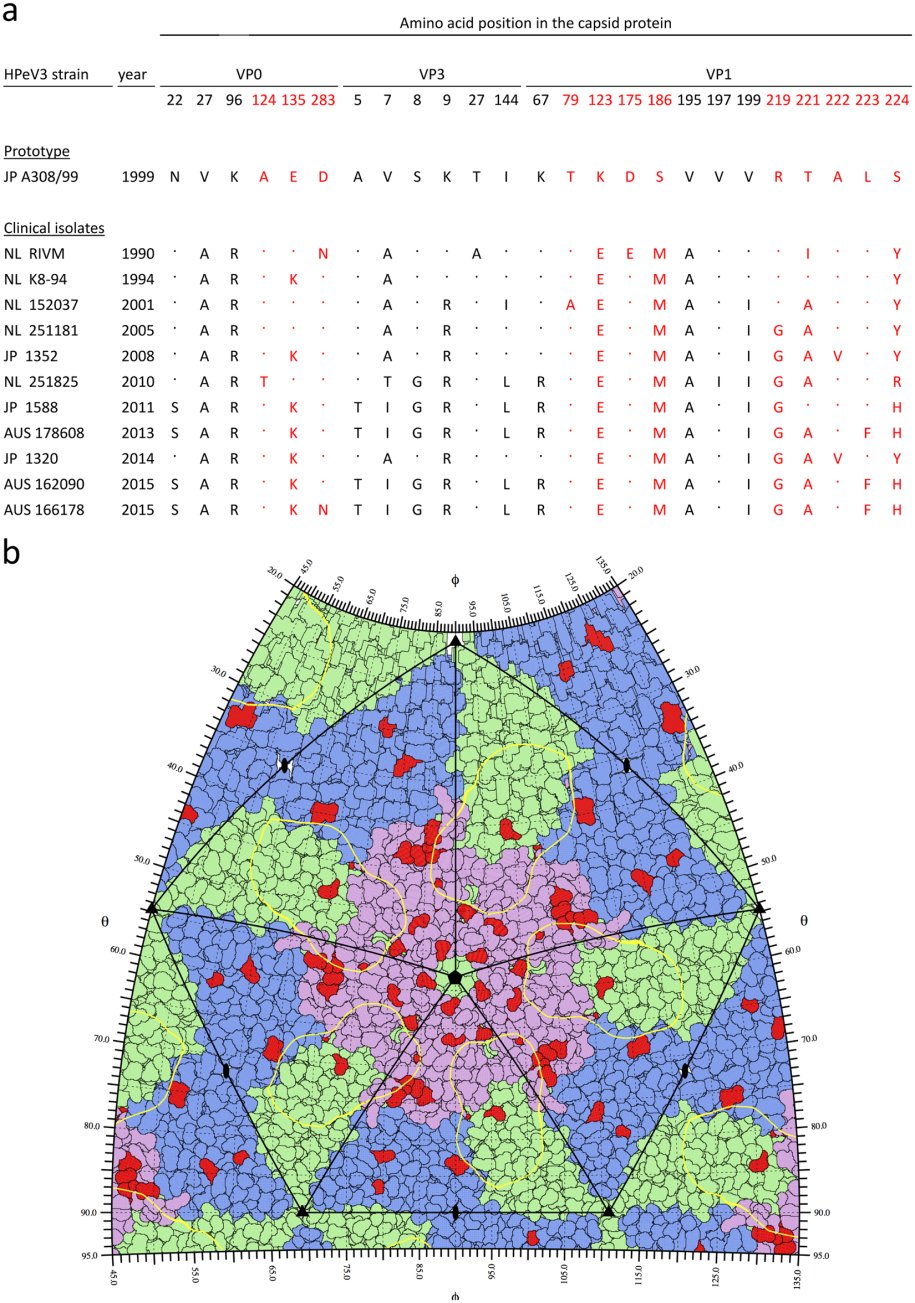
Amino acid variation in the capsid-encoding region of a subset of HPeV3 isolates. (**a**) Alignment showing the positions of variable amino acid residues. Capsid surface-exposed residues are highlighted in red. (**b**) HPeV3 roadmap of capsid exterior. Five full asymmetric units are shown; each formed of one copy of capsid proteins VP0 (blue), VP3 (green) and VP1 (pink). The AT12-015 Fab footprint (yellow contour) is mapped on surface residues differing between the strains (red), in comparison to the prototype A308/99. Residue-labeled close-up roadmaps for each strain are showed in Supplementary Information 1.

Capsid-encoding regions of a subset of HPeV3 isolates representing each geographical location and several points in time were sequenced in order to determine the genetic basis of the antigenic diversity. As surface-exposed amino acids on the capsid are accessible to host antibodies and likely to be antigenically most critical, we mapped the variable residues (Fig. 2a), and modelled these changes to the HPeV3 capsid structure (Supplementary Figs 1, 2 and 3). As shown in Fig. 2a, variable amino acid positions were detected in all capsid proteins, but mostly in VP1. Of the variable positions, three VP0 (124, 135 and 283) and nine VP1 (79, 123, 175, 186, 219 and 221–224) residues were surface-exposed as depicted in the HPeV3 roadmap image representing the exterior of an HPeV3 capsid pentamer (Fig. 2b), and in Supplementary Figures 1 and 2, which highlight the differences between the strains. Overall, non-synonymous changes were more numerous in the HPeV3 strains isolated post 2010 compared to the older clinical isolates or to the A308/99 prototype, and most of the amino acid variation in the capsid exterior was located in the VP1 C-terminus (Fig. 2a). We found a similar pattern of amino acid variation among HPeV3 VP1 sequences deposited in the GenBank (Supplementary Fig. 5).

### HPeV3 readily escapes neutralization by a human monoclonal antibody

**Figure 3:**
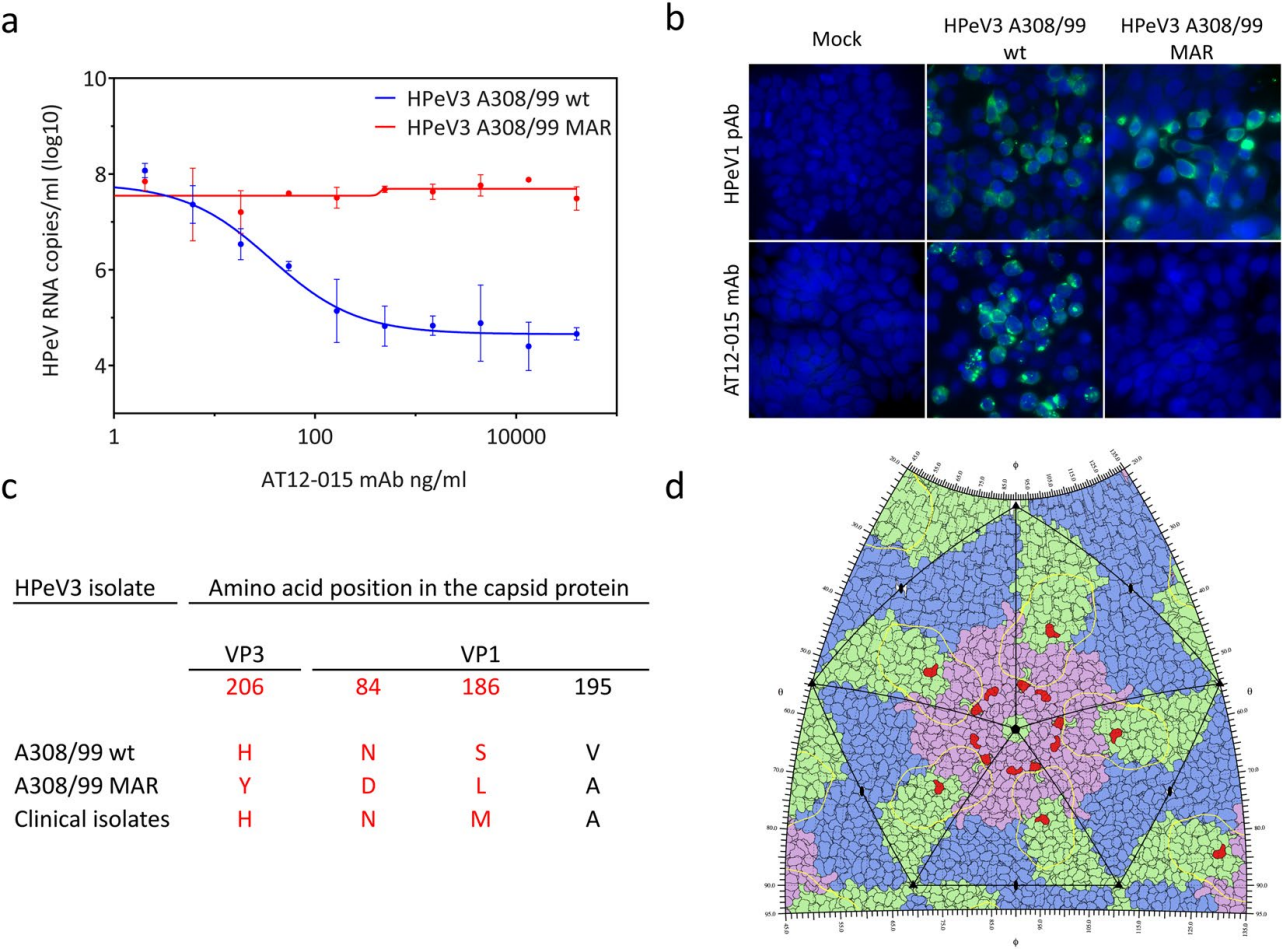
Characterization of the AT12-015 mAb resistant (MAR) HPeV3 variant. (**a**) Nonlinear regression analysis of the neutralizing activity of a human AT12-015 mAb against the HPeV3 A308/99 wild-type (wt) and the MAR HPeV3 variant. Data presented as the mean ± SD. (**b**) Immunofluorescence imaging of HPeV3 A308/99 wt and MAR variant infected HT29 cells stained by a rabbit HPeV1 antiserum (HPeV1 pAb) or the AT12-015 mAb. (**c**) Amino acid mutations in the MAR HPeV3 variant as compared to the A308/99 wt and the clinical isolates. Capsid surface-exposed residues are highlighted in red. (**d**) HPeV3 roadmap of capsid exterior. Capsid proteins VP0 (blue), VP3 (green) and VP1 (pink), the AT12-015 Fab footprint (yellow contour), and also amino acid mutations in variant resistant to AT12-015 are shown (red).

The mAb AT12-015 could only neutralize the HPeV3 A308/99 isolate (Fig. 1). The footprint, the surface area which the mAb AT12-015 covers on the virus capsid, was mapped to a conformational epitope in the canyon region of viral capsid proteins VP1 and VP3^28^. To determine which amino acids are involved in the escape from the AT12-015 mAb neutralization, we generated a mAb-resistant (MAR) HPeV3 variant by cultivating HPeV3 A308/99 wild type (wt) isolate in the presence of the mAb. As shown in Fig. 3a, the HPeV3 A308/99 wt was neutralized by the mAb with an IC_50_ of 36.1 ng/ml whereas the MAR HPeV3 variant could not be neutralized. Escape from neutralization by the mAb was confirmed in LLCMK2 cells (Supplementary Figure 4). In contrast the rabbit HPeV3 hyperimmune serum neutralized the MAR variant efficiently (Supplementary Figure 4). Immunostaining of HPeV3 A308/99 wt and the MAR variant in infected HT29 cells showed that mAb AT12-015 did not bind to the resistant variant (Fig. 3b). However, the mAb AT12-015 did bind the clinical isolates, which were also resistant to neutralization by this mAb (Supplementary Figure 4).

Sequence analysis of the capsid-encoding region of the AT12-015 MAR HPeV3 variant, revealed 4 mutations, VP3-H206Y, VP1-N84D, VP1-S186L, and VP1-V195A, as compared to A308/99 wt (Fig. 3c). Three of these mutated residues, VP3-H206Y, VP1-N84D, and VP1-S186L, are located on the capsid surface (Fig. 3d). However, only residue VP3-H206Y is located in the mAb AT12-015 footprint^28^. VP1-N84D is located close to the 5-fold annulus, residue VP1-S186L is located in a loop, and VP1-V195A is in a buried β-sheet close to the 5-fold annulus. We have previously seen that mAb AT12-015 does bind to but not neutralize strain 152037^28^. As residues VP1-186 and VP1-195 are conserved among the MAR variant and the clinical isolates, neither of these residues seem to be responsible for blocking the binding of mAb AT12-015 to the MAR variant. Although it is possible that the other mutations play a role, most likely the major contribution for preventing binding and thus neutralization comes from VP3-H206Y as it is located in the mAb AT12-015 footprint. None of the three surface-exposed amino acid variations are present in the clinical isolates and thus cannot explain the lack of neutralization of the clinical isolates sequenced for this study.

## Discussion

In this study we described antigenic diversity of HPeV3 strains isolated in various geographical locations and years. HPeV3 strains used in previous studies in the Netherlands and in Japan are antigenically distinct and we provided evidence that chloroform treatment can substantially influence the outcome of HPeV3 serological assays. These observations likely explain differences in the levels of HPeV3 nAb seropositivity reported in the literature.

Neutralizing antibody titers of the rabbit HPeV3 hyperimmune polyclonal serum as well as of the tested IVIG batches were considerably higher against the prototype HPeV3 A308/99 than against the clinical isolates, indicating that the prototype strain is an antigenically distinct outlier. We observed a decline in neutralization efficiency of the IVIG batches against HPeV3 strains isolated post 2005, including strains isolated during the HPeV3 epidemics in Japan and Australia^6,7,9,11,30^. As IVIG is composed of pooled IgG obtained from adult plasma donors, who may have not encountered recent HPeV3 strains, the loss of neutralization capacity against the circulating strains could reflect the exposure history of the plasma donors, and suggests antigenic drift. In line with this, we detected an increase in the number of non-synonymous mutations over time in the capsid-encoding region of our panel of HPeV3 isolates and found the majority of the surface-exposed amino acid variation in the VP1 protein. This is consistent with a report from Van der Sanden *et al*. which described VP1 amino acid variation in HPeV3 strains isolated in the Netherlands^20^. In HPeV1 the corresponding region contains the RGD motif and is highly immunogenic^26,27,31^. Similarly, antigenic determinants in VP1 near the receptor-binding sites have been described for other picornaviruses including enterovirus 71 (EV-A71), poliovirus (PV) and foot-and-mouth disease virus (FMDV)^32–34^. The HPeV3 VP1 C-terminus, which according to the structure is disordered but exposed on the surface^28^, may therefore contain antigenic sites of HPeV3. However, buried residues not exposed on the surface may also have a role in antigenicity via capsid breathing, as has been shown for an immunogenic epitope in the PV and enterovirus 70 VP1 protein^35,36^.

We recently isolated and characterized a human HPeV3-specific mAb (AT12-015), and found it incapable of neutralizing the HPeV3 152037 strain^28^. Here we showed that AT12-015 could bind to all the HPeV3 isolates tested but only neutralize the prototype HPeV3 A 308/99 strain. By generating a resistant variant, we found that four mutations in the capsid-encoding region are sufficient for escape from the mAb neutralization. The resistant variant, however, could be neutralized by the rabbit HPeV3 hyperimmune serum indicating presence of additional neutralizing epitopes. As AT12-015 mAb does not bind to the resistant variant, mutation of VP3-H206Y located within the previously mapped AT12-015 footprint is likely responsible for the escape from the mAb binding and neutralization with a potential contribution from VP1-N84D. However, this escape mechanism differs from that of the clinical isolates which are also resistant to neutralization by mAb AT12-015, but show capsid surface variation particularly in the VP1 C-terminus rather than in these three residues. As the VP1 C-terminus is predicted to be in the mAb footprint, variation in this region could affect neutralization. Therefore we speculate that the resistance of our panel of clinical HPeV3 isolates to the mAb could be due to two main reasons: (i) changes in the virion stability, i.e. compared to the clinical isolates the capsid of HPeV3 A308/99 is more stable upon mAb binding and does not disintegrate following entry, thus the genome is not released or (ii) differential receptor usage; meaning that mAb binding blocks the receptor binding site and entry of A308/99 but not that of other clinical isolates, including 152037.

Antigenic novelty in HPeV3 is generated by mutation of only a few amino acid residues on the capsid. Similar phenomena have been observed for other picornaviruses^13^. Genetic diversity of EV-A71 is associated with large outbreaks, and within-genotype antigenic variation of EV-A71 is known to occur^32,37^. In the case of PV, evidence of antigenically divergent strains of vaccine-derived PV has raised concerns about the final stages of PV eradication^33,38,39^. For EV-A71 and FMDV, it has been shown that instead of accumulating amino acid mutations across the entire capsid-encoding region, the antigenic evolution is driven by mutation of only a few key residues^32,40^.

Considerable variations in the nAb titers of rabbit hyperimmune polyclonal sera and of patient sera against different genogroups and subgenotypes of EV71 have been explained by antigenic diversity, patient and population exposure history as well as variation in cell lines^32,41^. Analogous to the related enterovirus echovirus 4^42^, we found a strain-dependent effect of chloroform treatment on HPeV3 neutralization. The HPeV3 prototype A308/99 strain, included in the studies showing high HPeV3 nAb seroprevalence in Japanese adults, did not require chloroform treatment for neutralization, whereas the Dutch HPeV3 152037 strain could only be neutralized following treatment. This implies that virus aggregation, or the presence of other chloroform-sensitive material, protects the Dutch strain against neutralization *in vitro* ^43^. The amino acid variation we observed in the HPeV3 capsid may have evolved in response to nAb pressure but we cannot exclude that it could partly be due to accumulation of neutral mutations, or virus passaging in cell culture as has been shown for FMDV^44^. Similarly, neutralization assays performed in different cell lines may explain the varying HPeV3 nAb seropositivity rates as reported by our group and others^15,19^. Future neutralization studies should include HPeV3 strains treated with chloroform, and IVIG batches from different geographical populations, varying in date of preparation, to confirm the occurrence of antigenic drift, and to assess the usefulness of IVIG infusion as a treatment for HPeV3-infected patients. In addition, further research, such as antigenic profiling by a panel of neutralizing mAbs and reverse genetics, is needed to identify the exact amino-acid residues which constitute the antigenic determinants of HPeV3.

In conclusion, we report here that the HPeV3 prototype strain A308/99 is antigenically distinct from all other isolates used in our study. Furthermore, we found majority of the surface-exposed amino acid variation in the VP1 protein and identified four amino acid mutations mediating HPeV3 escape from a neutralizing human mAb. Antigenic variation of HPeV3 can promote the persistence of HPeV3 in the human population and explain the escape from protection by maternal Abs in neonates as well as facilitate the regularly occurring outbreaks. Further characterization of the divergent HPeV3 strains will enable identification of epitopes as targets for the design of therapeutic antibodies.

## Methods

### HPeV3 isolates and cell lines

25 HPeV3 strains isolated from patient samples in Europe, Asia and Australia over a period of three decades were included in this study (Table 1). The HPeV3 A380/99 prototype strain was provided by the National Institute of Infectious Diseases, Tokyo, Japan. Dutch HPeV3 strains were isolated as part of primary diagnostics at the Academic Medical Center (AMC) in Amsterdam between 1994 and 2010, and as part of the national enterovirus surveillance in 1989 and 1990 at the National Institute of Public Health and the Environment (RIVM; Bilthoven). Other strains were obtained from the Victorian Infectious Diseases Reference Laboratory, Doherty Institute (VIDRL; Melbourne, Australia) and the Yamagata Prefectural Institute of Public Health (YPIPH; Yamagata, Japan) and were isolated between 2008 and 2015. Virus isolates included in the standard neutralization assay were plaque purified and amplified in LLCMK2 cells (rhesus monkey kidney cell line, kindly provided by the Municipal Health Services, Rotterdam, the Netherlands) maintained in Eagle’s minimum essential medium (EMEM; Lonza, Basel, Switzerland) supplemented with 10% heat-inactivated fetal bovine serum (FBS; Sigma-Aldrich, St. Louis, MO), streptomycin (100 µg/ml; Lonza Bio Whittaker), penicillin (100U/ml; Lonza Bio Whittaker), non-essential amino acids (NEAA; ScienCell Research Laboratories, Carlsbad, CA) and L-glutamine (200 nM; Lonza, Basel, Switzerland). Following plaque purification, all isolates were genotyped using HPeV VP1-specific primers (Supplementary Table 1., RIVM; Bilthoven). Chloroform treatment of the virus stocks was performed as follows; 10% (v/v) chloroform (Sigma-Aldrich, St. Louis, MO) was added to each virus culture and vortexed vigorously for 5 minutes. Chloroform was removed by centrifugation for 10 minutes at 3000 rpm. The 50% cell culture infective dose (CCID_50_) of virus stocks was determined by means of end point dilution. Briefly, in a 96-well format, 8 replicates of a 10-fold serial dilution of the stocks were incubated with LLCMK2 cells in EMEM supplemented with 10% FBS at 37 °C, 5% CO_2_ for 7 days. The CCID_50_ was calculated by scoring CPE in all wells and using the Reed and Muench formula^45^. HT29 cells (human colorectal adenocarcinoma; ATCC, Manassas, VA), used to study HPeV3 mAb resistance, were maintained in McCoy’s 5 A medium (GE Healthcare HyClone, Chicago, IL) supplemented with 2% heat-inactivated FBS, streptomycin (100 µg/ml) and penicillin (100U/ml). The studies described here were performed in accordance with relevant guidelines and regulations, and the AMC Research Code (https://www.amc.nl/web/AMC-website/Research-Code/1-Introduction.htm).

**Table 1:** List of HPeV3 isolates included in this study.

HPeV3 isolate	Year	Origin	Accession
JP A308/99 (prototype)	1999	NIID	AB084913
NL RIVM 1989	1989	RIVM	na
NL RIVM 1990	1990	RIVM	na
NL K8-94	1994	AMC	GQ183033
NL K11-94	1994	AMC	GQ183030
NL K12-94	1994	AMC	GQ183031
NL K20-94	1994	AMC	GQ183032
NL 152037	2001	AMC	GQ183026
NL 451371	2004	AMC	DQ172449
NL 251181	2005	AMC	DQ172443
NL 251825	2010	AMC	na
JP 1352	2008	YPIPH	AB759185
JP 1443	2008	YPIPH	AB668032
JP 1448	2008	YPIPH	AB668033
JP 1518	2008	YPIPH	AB759189
JP 1384	2011	YPIPH	AB759148
JP 1588	2011	YPIPH	AB759194
JP 1615	2014	YPIPH	LC043128
JP 1557	2014	YPIPH	LC043127
JP 1320	2014	YPIPH	LC043114
AUS 178608	2013	VIDRL	na
AUS 158108	2015	VIDRL	na
AUS 162090	2015	VIDRL	na
AUS 166507	2015	VIDRL	na
AUS 166178	2015	VIDRL	na

National Institute of Infectious Diseases (NIID, Japan) Rijksinstituut voor Volksgezondheid en Milieu (RIVM, the Netherlands), Academic Medical Center (AMC, the Netherlands), Yamagata Prefectural Institute of Public Health (YPIPH, Japan), Victorian Infectious Diseases Reference Laboratory, Doherty Institute (VIDRL, Australia). Accession numbers are indicated if available (na; not available).

### IVIG batches and antibodies

Two intravenous immunoglobulin (IVIG) batches collected from the Dutch population (manufactured in 2008 and 2010) were obtained from Nanogam (Sanquin, Amsterdam, the Netherlands) and the two Japanese IVIG batches were from Takeda Pharmaceutical (Osaka, Japan; year of manufacture unknown) and Teijin Pharma (Osaka, Japan; year of manufacture prior to August 2013). Rabbit polyclonal serum raised against the Dutch HPeV3 152037 strain was produced by Harlan Laboratories (Loughborough, UK). An HPeV3-specific human monoclonal antibody AT12-015 was isolated by AIMM Therapeutics (Amsterdam, the Netherlands) in 2012 from an HPeV3-infected Dutch adult (Karelehto *et al*., manuscript in preparation). The footprint of this mAb was recently mapped on the HPeV3 152037 strain capsid structure^28^.

### *In vitro* neutralization assay

*In vitro* endpoint neutralization assays were performed as follows. A twofold serial dilution of the sera and IVIG preparations was incubated with an equal volume of chloroform-treated 100 CCID_50_ virus in duplicates at 37 °C, 5% CO_2_, for 1 hour. LLCMK2 cells in EMEM supplemented with 10% FBS were subsequently added and plates were incubated at 37 °C, 5% CO_2_ for 7 days. The neutralizing titer was calculated on the basis of the number of wells showing cytopathogenic effect (CPE) using the Reed and Muench method and reported as the reciprocal titers of serum dilutions that exhibited 50% neutralization^45^. A neutralizing titer of ≥ 5.0 log2 was used as a threshold for nAb seropositivity as it has been correlated with protection against disease, and used in recently published reports^19,23^. HPeV3 isolate group median nAb titers with interquartile range were determined and compared by Kruskal-Wallis test with Dunn’s post hoc analysis (significance level p < 0.05) in GraphPad Prism 7 (GraphPad Software Inc., La Jolla, CA).

### Sequence analyses

RNA from selected HPeV3 isolate stocks was extracted using the Boom method^46^ and reverse transcribed as described previously^47^. Seven primer pairs were used to PCR amplify overlapping fragments of the capsid-encoding region of HPeV3 (Supplementary Table 2). The amplicons were sequenced using the BigDye Terminator Cycle Sequencing Ready Reaction Kit on an automated sequencer (Applied Biosystems, Foster City, CA). Sequence assemblies were generated with CodonCode Aligner 6.0.2 (CodonCode Corporation, Dedham, MA) and aligned with the ClustalW method in BioEdit 7.0.9.0^48^. The nucleotide sequences generated in this work are deposited in GenBank under accession numbers KY930873-KY930884.

### Generation of AT12-015 monoclonal antibody resistant HPeV3 variant

An AT12-015 monoclonal antibody resistant (MAR) HPeV3 variant was generated by a clonal selection protocol as described previously^49^. Briefly, HT29 cells in 96-well plate microtiter plates were inoculated with a serial dilution of the HPeV3 prototype A308/99 strain and cultured in the presence of serially diluted (0.0007–120.0 µg/ml) HPeV3 monoclonal antibody AT12-015 (AIMM Therapeutics)^28^. Conditions with minimal virus input producing CPE within 7 days post-inoculation and the lowest mAb concentration (234 ng/ml) which completely inhibited formation of the CPE within this time were used to prepare six 96-well plates of infected cells. Cells were monitored daily. At day 10 post-inoculation CPE was observed in a single well out of 396 identical conditions and the cells and media were harvested. The AT12-015 mAb resistant strain was amplified in HT29 cells in the presence of the mAb (234ng/ml).

### AT12-015 monoclonal antibody IC50 determination

100 CCID_50_ of the wild-type (wt) HPeV3 A308/99 prototype strain or the AT12-015 MAR HPeV3 variant were preincubated with serially diluted concentrations of the AT12-015 mAb at 37 °C, 5% CO_2_ for 1 hour and used to inoculate monolayers of HT29 cells in 96-well microtiter plates in duplicates. At day 7 post-inoculation, 25 µl of the culture supernatants were lysed by addition of 25 µl of Cells-to-cDNA™ II Cell Lysis Buffer (ThermoFisher Scientific, Waltham, MA) and incubated at +72 °C for 15 minutes. HPeV genomic RNA was quantified from 5 µl of each lysed sample using detection primers and a probe described previously^47^ and a TaqMan One-Step RT-PCR kit (Applied Biosystems). Copy numbers of HPeV genomes in the medium samples were quantified using a standard curve, which was generated by performing the RT-PCR on serial dilutions of a previously constructed control plasmid containing an HPeV amplicon^47^. Half maximal inhibitory concentrations (IC_50_) were determined by nonlinear regression analysis performed in GraphPad Prism 7.

### Immunofluorescence imaging

HT29 cells were cultured on cover slips in a 24-well microtiter plate and infected with the wt HPeV3 prototype A308/99 strain or the AT12-015 MAR HPeV3 variant at a multiplicity of infection (MOI) of 5. LLCMK2 cells were cultured on cover slips in a 24-well microtiter plate and infected at a MOI of 0.3 with one the following clinical isolates; NL RIVM 1990, NL K8-94, NL 152037, JP 1352, NL 251825, JP 1588, JP 1320 and AUS 162090. HT29 cells were fixed 24 hours and LLCMK2 cells 48 hours post-inoculation with 4% formalin and permeabilized with 0.2% Triton X-100. Cells were stained either with rabbit HPeV1 hyperimmune polyclonal serum (kindly provided by Dr. Susi, University of Turku, Finland^26^) previously determined to cross-react with HPeV3 (data not shown) or with the mAb AT12-015. Secondary antibodies used were donkey anti-rabbit IgG Alexa488 (ThermoFisher Scientific) or goat anti-human IgG Alexa488 (ThermoFisher Scientific). Cover slips were mounted on objective slides using ProLong Gold Antifade Mountant with DAPI (ThermoFisher Scientific) and images were acquired using Leica DM-RA microscope with PLAN APO 100x/1.40/oil Phaco3 objective (Leica Microsystems, Wetzlar, Germany).

### 2D roadmap generation

First, predictions of the full structure of HPeV3 A308/99 VP0, VP1 & VP3 capsid proteins including unstructured regions were obtained by multiple-template homology modeling using the I-TASSER server^50^, with PDB:4Z92^51^ as the supplied primary template. The homology models of the VPs were then combined based on PDB:5APM^28^ to form the asymmetric unit, using MatchMaker in Chimera^52,53^. Subsequently, a pseudoatomic model of the whole icosahedral capsid was composed: no clashes were observed between the homology modeled regions and other chains. Roadmaps of the capsid exterior were generated using RIVEM^54^, with differences between strains shown by labeling residues and changing their displayed color. Footprint of the mAb, denoted by a contour, is calculated from EMDB:3138 for radius 161 Å, at the average map density^28^.

### 3D visualization of residue changes

For each strain, structured residues in the modeled HPeV3 A308/99 asymmetric unit that differed from the strain sequence were exchanged in Chimera with rotamers selected from the Dunbrack Rotamer Library 2002^55^, minimizing clashes with the whole capsid atomic model. After charge assignment^56^, all residues within 5 Å of replaced residues were subjected to energy minimization using 100 steepest-gradient steps and 10 conjugate gradient steps, using an AMBER ff14sb force field^57^. 3D visualization was performed in Chimera.

### Data Availability

All data generated or analyzed during this study are included in this published article (and its Supplementary Information files).

## Electronic supplementary material

Supplementary information
