# Supplementary material for: Strain-dependent neutralization reveals antigenic variation of human parechovirus 3
Source: Sci Rep. 2017 Sep 21;7:12075. doi: 10.1038/s41598-017-12458-5 (PMC5608956; doi:10.1038/s41598-017-12458-5)
Supplement: Supplementary file 1 — Supplementary information [file 41598_2017_12458_MOESM1_ESM.pdf]

Supplementary Information

Title

Strain-dependent neutralization reveals antigenic variation of human parechovirus 3

Authors

Eveliina Karelehto[1*], Sabine van der Sanden[1], James A Geraets[2], Aušra Domanska[2], Lonneke van der Linden[1], Dionne Hoogendoorn[1], Gerrit Koen[1], Hetty van Eijk[1], Shabih Shakeel[2], Tim Beaumont[3], Menno de Jong[1], Dasja Pajkrt[4], Sarah J Butcher[2] and Katja C Wolthers[1]

[1]Department of Medical Microbiology, Laboratory of Clinical Virology, Academic Medical Center, University of Amsterdam, The Netherlands
[2]Institute of Biotechnology & Department of Biosciences, University of Helsinki, Finland
[3]AIMM Therapeutics, Academic Medical Center, Amsterdam, the Netherlands
[4]Department of Pediatric Infectious Diseases, Emma Children's Hospital, Academic Medical Center (AMC), Amsterdam, the Netherlands

*Corresponding author

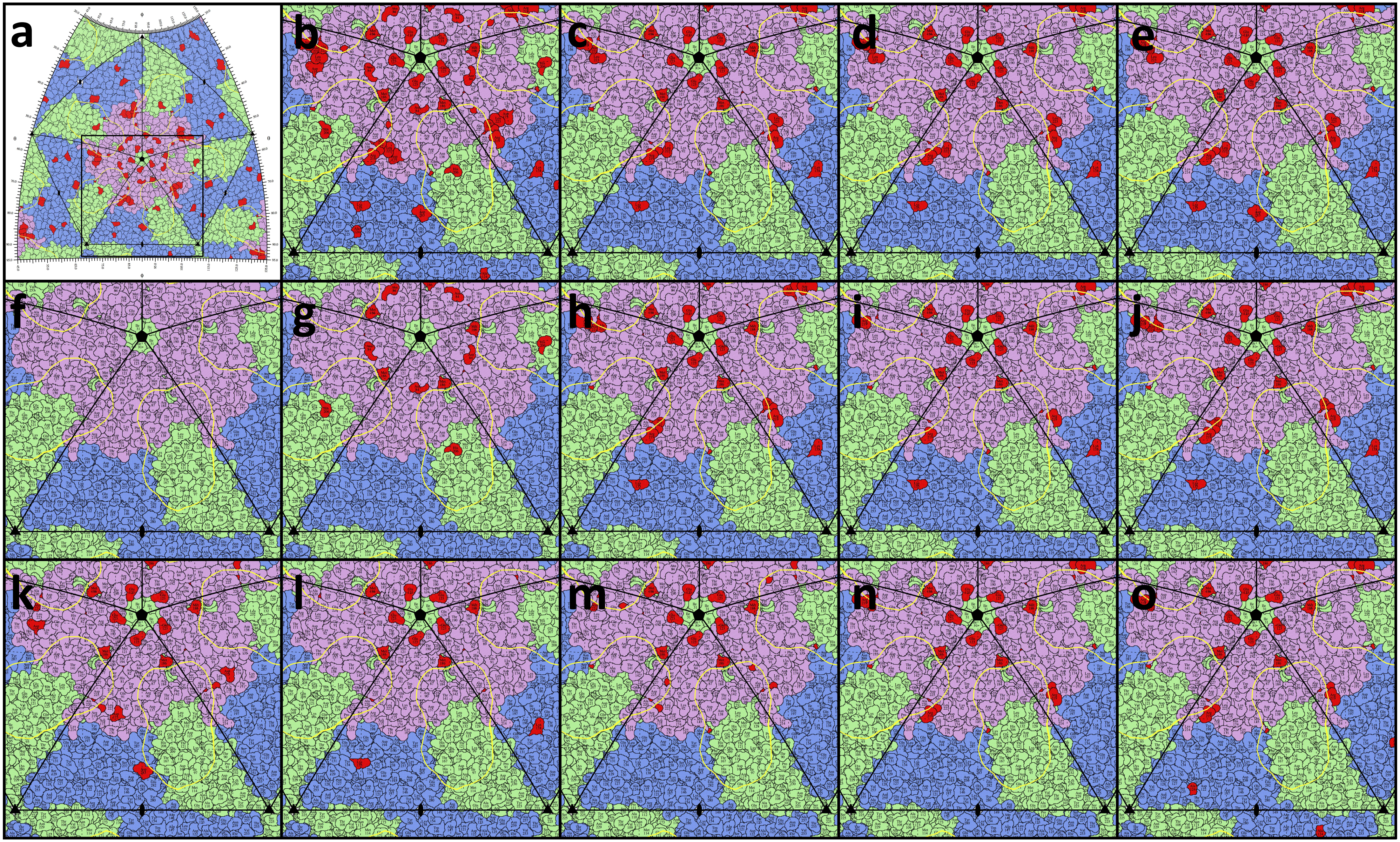

Supplementary Figure 1. Labeled roadmaps of HPeV3 strains in comparison to the prototype A308/99, showing capsid proteins VP0 (blue), VP3 (green) and VP1 (pink). The AT12-015 Fab footprint (yellow contour) is mapped on annotated surface residues differing between the strains (red). a) Enlarged view showing all surface variation for this subset of HPeV3 isolates, with amino acid residues colored red. b) Close-up labeled view of surface variation. c-o) Comparison of individual strains to A308/99 prototype: c) AUS 178608 2013. d) AUS 162090 2015. e) AUS 166178 2015. f) JP A308/99 prototype WT. g) JP A308/99 resistant variant. h) JP 1352 2008. i) JP 1588 2011. j) JP 1320 2014. k) NL RIVM 1990. l) NL K8-94 1994. m) NL 152037 2001. n) NL 2051181 2005. o) NL 21051825 2010.

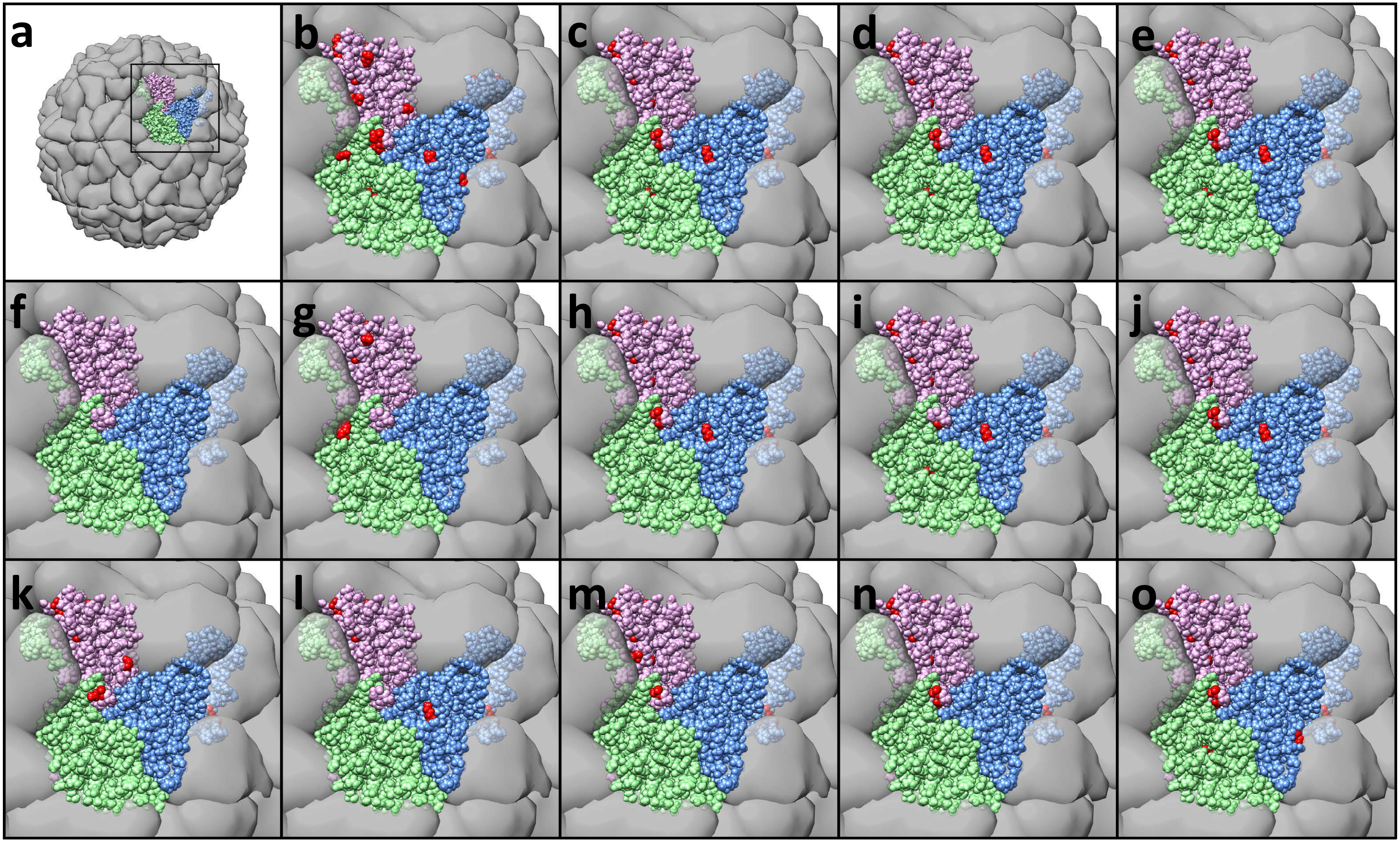

Supplementary Figure 2. Modeling of surface differences between HPeV3 strains. a) Position of modeled asymmetric unit of HPeV3 A308/99 in the capsid, showing capsid proteins VP0 (blue), VP3 (green) and VP1 (pink) (other protein units are partially transparent). b) Close-up view of asymmetric unit with all variation in amino acid residues colored red (not minimized). c-o) Differences in amino acid residues between HPeV3 strains compared to prototype A308/99 (red), modeled as described in Materials and Methods: c) AUS 178608 2013. d) AUS 162090 2015. e) AUS 166178 2015. f) JP A308/99 prototype WT. g) JP A308/99 resistant variant. h) JP 1352 2008. i) JP 1588 2011. j) JP 1320 2014. k) NL RIVM 1990. l) NL K8-94 1994. m) NL 152037 2001. n) NL 2051181 2005. o) NL 21051825 2010.

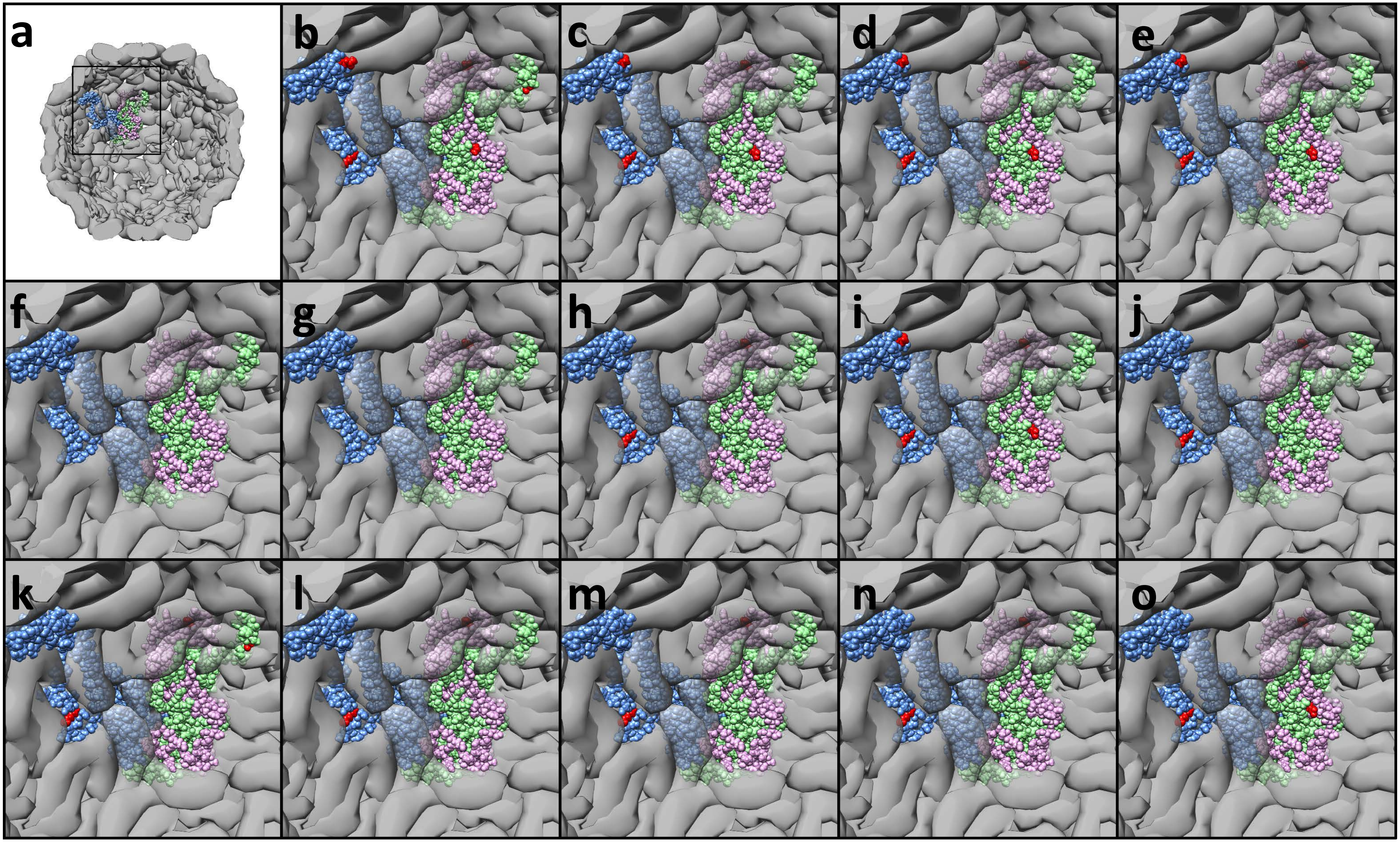

Supplementary Figure 3. Modeling differences in the capsid interior between HPeV3 strains. a) Position of asymmetric unit of HPeV3 in a rear slice of the capsid, showing capsid proteins VP0 (blue), VP3 (green) and VP1 (pink) (other protein units are partially transparent). b) Close-up view of asymmetric unit with all variation in amino acid residues colored red (not minimized). c-o) Differences in amino acid residues between HPeV3 strains compared to prototype A308/99 (red), modeled as described in Materials and Methods: c) AUS 178608 2013. d) AUS 162090 2015. e) AUS 166178 2015. f) JP A308/99 prototype WT. g) JP A308/99 resistant variant. h) JP 1352 2008. i) JP 1588 2011. j) JP 1320 2014. k) NL RIVM 1990. l) NL K8-94 1994. m) NL 152037 2001. n) NL 2051181 2005. o) NL 21051825 2010.

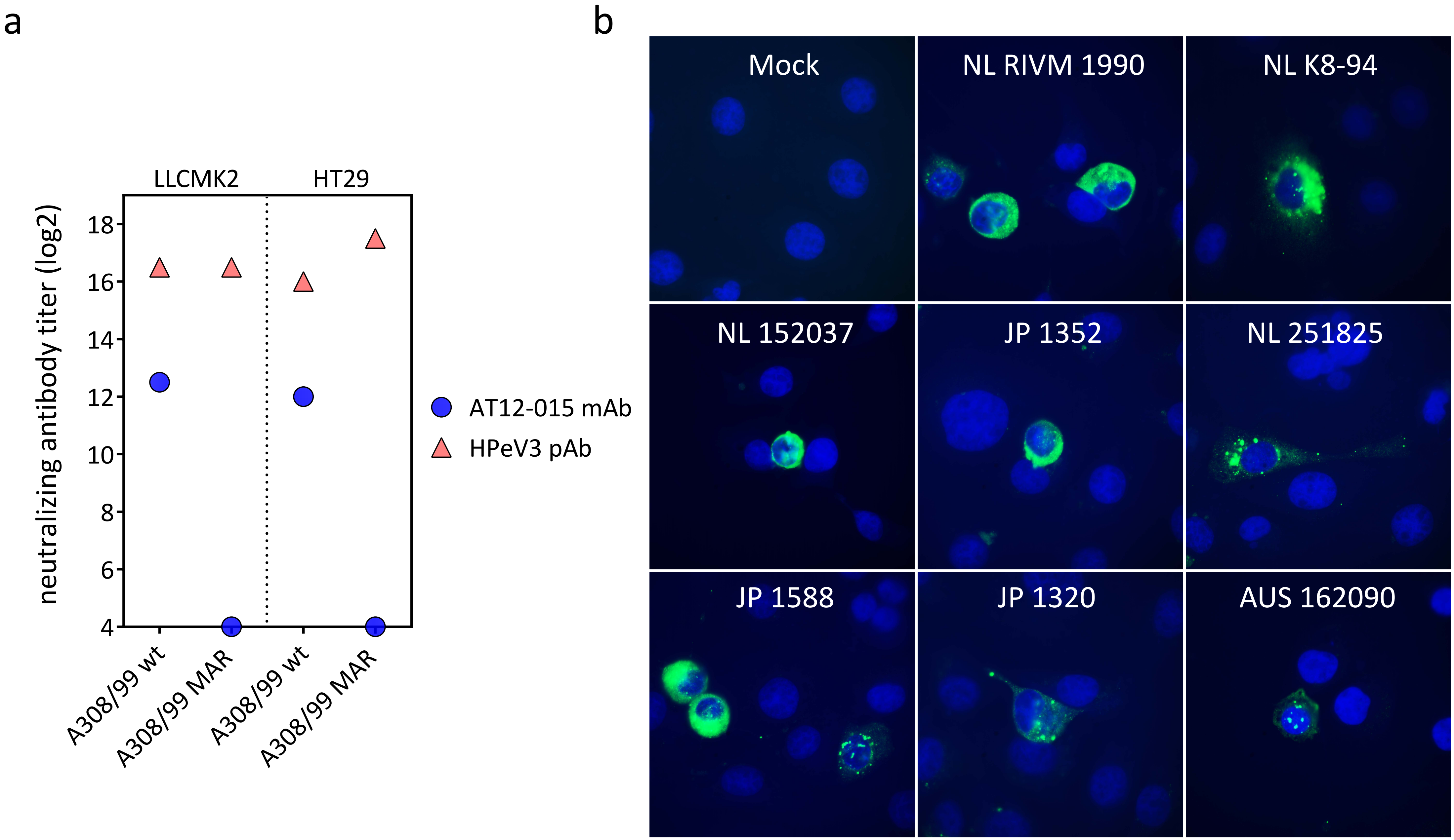

Supplementary Figure 4a) Analysis of the neutralizing activity of a human AT12-015 mAb and a rabbit HPeV3 hyperimmune polyclonal serum (HPeV3 pAb) against chloroform-treated HPeV3 A308/99 wild-type (wt) and MAR HPeV3 variant in LLCMK2 and HT29 cells. b) Immunofluorescence imaging of LLCMK2 cells infected with a subset of HPeV3 clinical isolates and stained by the AT12-015 mAb.

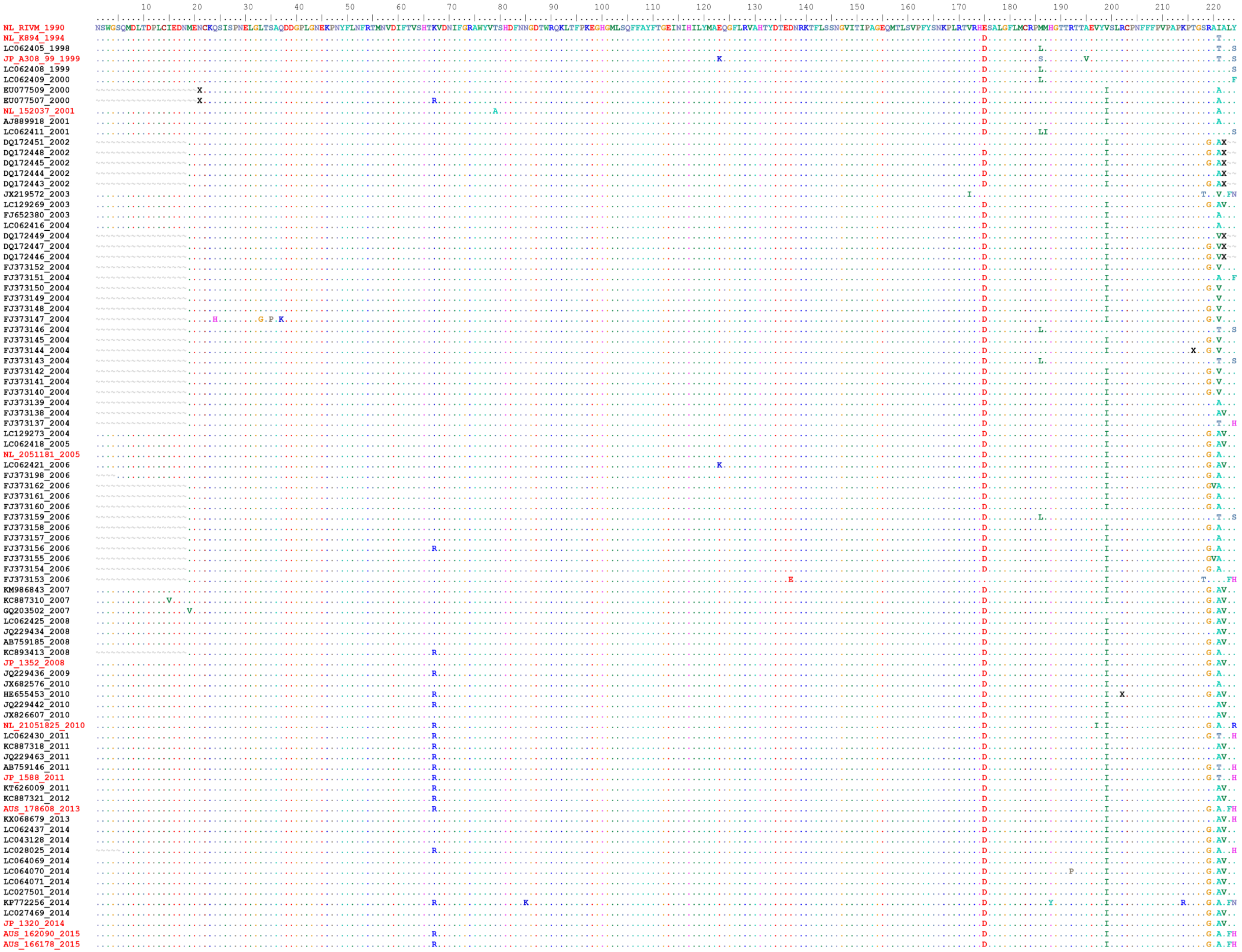

Supplementary Figure 5. Alignment of HPeV3 VP1 amino acid sequences from GenBank. HPeV3 strains are identified by the accession numbers and the year of isolation. Sequences generated in this study are highlighted in red.

Supplementary table 1. HPeV genotyping primers (RIVM).

#	Forward, 5′– 3′	Reverse, 5′–3′
1	TNMGNATGGGNTTYTTYCCNAAY	ARTARTCNARYTCRCAYTCYTC
2	GAGTTGGACAATGCCATCTAYACNATNTGYG	GTTCCTGTTAGAGCTGTCTTRAAnATRTCRTC

Supplementary table 2. HPeV3 P1 sequencing primers.

#	Forward, 5′– 3′	Reverse, 5′–3′
1	CCGTAGGTAACAAGTGRC	CAGTTCCTTGGTTGACRTT
2	GGAGACAARAACCCCAGTAA	CYGAAAGAGGYGTCCAAACA
3	CTAGGCAGTACRAAATAACC	ACTACTACTTGAAGGTGTGG
4	CTAATGAGGTTGATGTYACAGT	GAGAAGGARTATGGTATAGTCAA
5	CAATCTGTGGCTTCAGTGG	GTCAACATTCATAGTTCTGAAG
6	CACTTGGATGAGGAAGACACA	AAGRAACCCCTGCTYTGCC
7	CCKTTGGGTAATGAGAAACCA	AAYTGATATACAYTMTCTCCTA
